# P-47. Vaccine Effectiveness Against Influenza-Associated Hospitalizations in Adults with Liver Diseases

**DOI:** 10.1093/ofid/ofae631.254

**Published:** 2025-01-29

**Authors:** Po-Han Huang, Mary Patricia Nowalk, Richard K Zimmerman, Samantha M Olson, Keipp Talbot, Yuwei Zhu, Manjusha Gaglani, Kempapura Murthy, Arnold Monto, Emily T Martin, Fernanda Silveira, G K Balasubramani

**Affiliations:** School of Public Health, University of Pittsburgh, Pittsburgh, Pennsylvania; University of Pittsburgh, Pittsburgh, PA; University of Pittsburgh, Pittsburgh, PA; Centers for Disease Control and Prevention, Atlanta, Georgia; Vanderbilt University Medical Center, Nashville, Tennessee; Vanderbilt University, Nashville, Tennessee; Baylor Scott & White Health, Temple, TX; Baylor Scott and White Health, Temple, Texas; University of Michigan, Ann Arbor, MI; University of Michigan, Ann Arbor, MI; University of Pittsburgh, Pittsburgh, PA; University of Pittsburgh, Pittsburgh, PA

## Abstract

**Background:**

Influenza causes 100,000-710,000 hospitalizations annually in the U.S. Patients with liver disease are at higher risk of severe outcomes following influenza virus infection. While vaccination has been shown to be effective in preventing morbidity and mortality, patients with liver disease experience immune dysfunction that may attenuate influenza vaccine effectiveness (VE). Limited influenza VE data for this population are available. This study evaluated VE against influenza-associated hospitalization among adults with liver disease.

**Figure:**
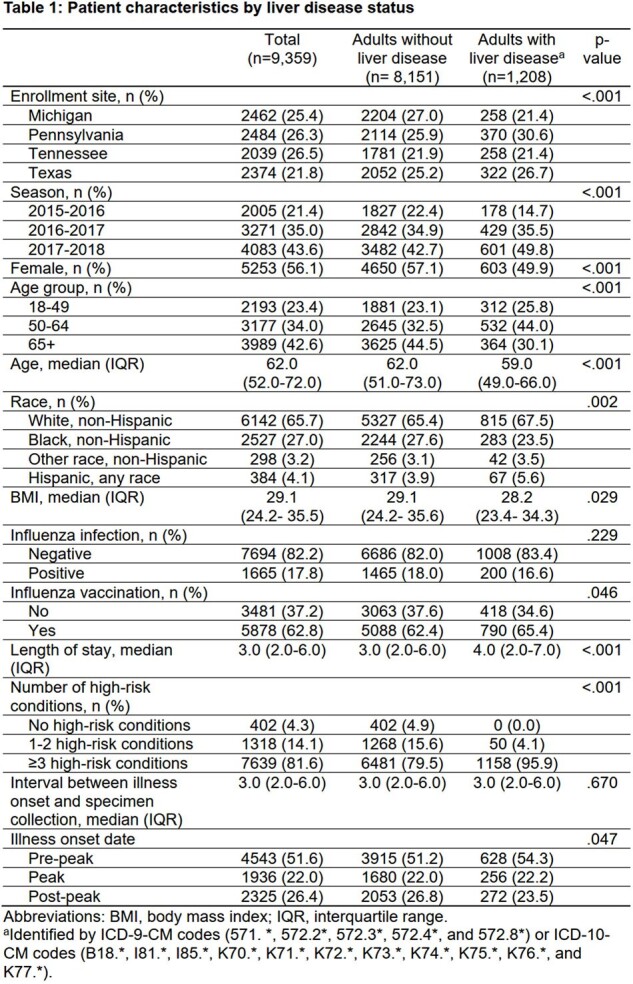

**Methods:**

We used a test-negative, case-control study to estimate VE among adults ≥18 years admitted at four sites within the U.S. Hospitalized Adult Influenza Vaccine Effectiveness Network (HAIVEN) for acute respiratory illness (ARI) during 3 influenza seasons (2015-2018). Adults with liver disease were identified by ICD-9/ICD-10-CM codes recorded ≤1 year before enrollment. VE was defined as (1-adjusted odds ratio) *100 by comparing the odds of having laboratory-confirmed influenza-associated hospitalization between vaccinated and non-vaccinated adults using multiple logistic regression models that incorporated inverse probability of treatment weighting with propensity score.

**Figure:**
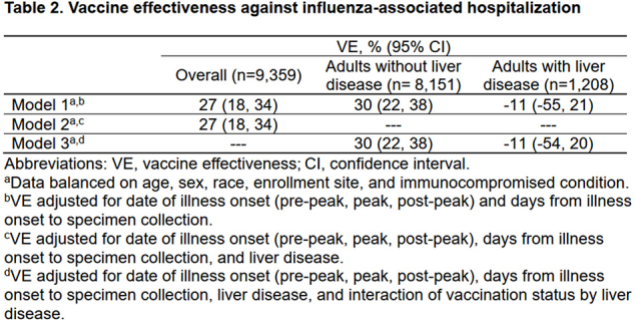

**Results:**

In total, 1208 (12.9%) of 9359 adults hospitalized for ARI had ≥1 liver disease(s). Compared with those without liver disease, adults with liver disease were slightly more likely to have received influenza vaccine (65.4% vs 62.4%; *p* = 0.046) but not different in their likelihood of influenza illness (16.6% vs 18.0%; *p* = 0.229). Overall, VE against influenza-associated hospitalization was 27% (95% confidence interval [CI], 18-34%); 30% (95% CI, 22-38%) among patients without liver disease; and -11% (95% CI, -54% to 20%) among those with liver disease. Significant effect modification of VE by the presence of liver disease was found (*p* = 0.01 for interaction term).

**Figure:**
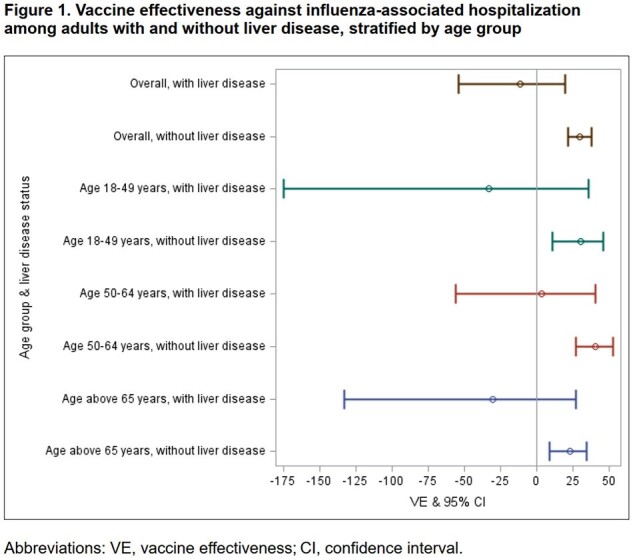

**Conclusion:**

We observed no association of influenza vaccination on influenza-associated hospitalizations among adults with liver disease. Further studies are warranted to evaluate VE in patients with different types of liver disease to better understand if effectiveness varies by specific liver diseases and to investigate potential residual confounding.

**Figure:**
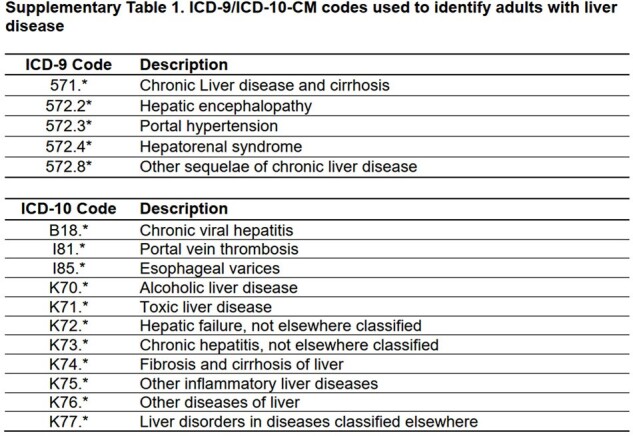

**Disclosures:**

**Po-Han Huang, MD, MSc**, Pfizer: Honoraria **Mary Patricia Nowalk, PhD**, AstraZeneca - Icosavax: Grant/Research Support|GSK: Advisor/Consultant|Merck & Co.: Grant/Research Support|Sanofi: Grant/Research Support **Richard K. Zimmerman, MA;MD;MPH;MS**, Sanofi Pasteur: Grant/Research Support **Arnold Monto, MD**, Roche: Advisor/Consultant **Fernanda Silveira, MD**, Ansun: Grant/Research Support|GSK: Grant/Research Support|Merck: Grant/Research Support|Viracor Eurofins: Advisor/Consultant

